# Prediction of Antimicrobial Peptides Based on Sequence Alignment and Feature Selection Methods

**DOI:** 10.1371/journal.pone.0018476

**Published:** 2011-04-13

**Authors:** Ping Wang, Lele Hu, Guiyou Liu, Nan Jiang, Xiaoyun Chen, Jianyong Xu, Wen Zheng, Li Li, Ming Tan, Zugen Chen, Hui Song, Yu-Dong Cai, Kuo-Chen Chou

**Affiliations:** 1 Tianjin Institute of Industrial Biotechnology, Chinese Academy of Sciences, Tianjin, China; 2 Institute of Systems Biology, Shanghai University, Shanghai, China; 3 Department of Chemistry, College of Sciences, Shanghai University, Shanghai, China; 4 Department of Human Genetics, University of California Los Angeles, Los Angeles, California, United States of America; 5 Gordon Life Science Institute, San Diego, California, United States of America; University of South Florida College of Medicine, United States of America

## Abstract

Antimicrobial peptides (AMPs) represent a class of natural peptides that form a part of the innate immune system, and this kind of ‘nature's antibiotics’ is quite promising for solving the problem of increasing antibiotic resistance. In view of this, it is highly desired to develop an effective computational method for accurately predicting novel AMPs because it can provide us with more candidates and useful insights for drug design. In this study, a new method for predicting AMPs was implemented by integrating the sequence alignment method and the feature selection method. It was observed that, the overall jackknife success rate by the new predictor on a newly constructed benchmark dataset was over 80.23%, and the Mathews correlation coefficient is 0.73, indicating a good prediction. Moreover, it is indicated by an in-depth feature analysis that the results are quite consistent with the previously known knowledge that some amino acids are preferential in AMPs and that these amino acids do play an important role for the antimicrobial activity. For the convenience of most experimental scientists who want to use the prediction method without the interest to follow the mathematical details, a user-friendly web-server is provided at http://amp.biosino.org/.

## Introduction

Natural gene-encoded antimicrobial peptides (AMPs) are a group of small, innate immune molecules, generally containing 12–100 amino acid residues [1]. AMPs have been discovered in most life forms, including bacteriocins, fungal peptide antibiotics, plant thionins and defensins, insect defensins and cecropins, amphibian magainins and temporis, as well as defensins and cathelicidins from higher vertebrates [1], [2], [3]. Owing to the broad spectrum antimicrobial activity [4], [5], antibacteria, antifungi, antivirus, and even anticancer, are thought to be less likely to induce resistance. Thus, AMPs have attracted the attention of many investigators as a substitute for conventional antibiotics [1]. Currently, most researchers in this area are focused on screening and in silico modeling novel AMPs [6], [7] as computational approaches can accelerate the process of antimicrobial drug discovery and design [8]. Many bioinformatics methods have been developed for predicting new AMPs. For example, the APD method predicted whether the new peptide had the potential to be antimicrobial based on some known principles [9]. The AMPer method [10] was developed by constructing the hidden Markov models (HMMs) to automatically discover AMPs. The BACTIBASE [11], [12] and PhytAMP [13] methods were specifically designed for bacteriocin and plant respectively. The AntiBP method [14] and AntiBP2 method [15] used the Artificial Neural Network (ANN), Quantitative Matrices (QM) and Support Vector Machine (SVM) to predict antibacterial peptides. Their training sets were limited to N and/or C terminus residues of peptides. The CAMP method [16] was developed based on the Random Forests (RF), SVM, and Discriminant Analysis (DA), trained on all classes of AMPs (antibacterial, antifungal and antiviral) and full length of mature AMP sequences. However, none of the aforementioned methods has the function to identify which kinds of features are optimal for accurately predicting and meaningfully interpreting their biological implications.

The present study was initiated in an attempt to establish a new classification method for predicting AMPs by integrating the sequence alignment method and the feature selection method. In the sequence alignment method, the prediction was carried out by assigning the query peptide to the category of the peptide that has the highest sequence similarity with the query peptide. In the feature selection method, each peptide was coded with 270 features, including amino acid composition [17], [18] and pseudo-amino acid composition [19] that incorporated electrostatic charge, codon diversity, molecular volume, polarity, and secondary structure [20]. Subsequently, the feature selection and analysis methods, including the Maximum Relevance Minimum Redundancy method (mRMR) [21] and the Incremental Feature Selection (IFS) [22] method, were employed to select the optimal features for the prediction of AMPs versus non-AMPs. The prediction model was built using the well-known Nearest Neighbor Algorithm (NNA) [23], [24], [25]. As a result, the methods achieved a satisfactory overall success rate.

## Materials and Methods

### Datasets

#### Training set

The AMP sequences were downloaded from CAMP [16]. The 1,216 AMP sequences validated by experiments and the 1,651 AMP sequences filed with patents were used. After eliminating those sequences with non-standard residues ‘B’, ‘J’, ‘O’, ‘U’, ‘X’, or ‘Z’, the final positive dataset contained 2,752 AMP sequences, of which only 35 peptides in UniPort database [26], [27] are annotated with experimentally-verified no antimicrobial activity. Because AMPs are generally secretory in nature [28], we also randomly selected 10,000 non-secretory protein sequences from UniProt database without annotated by ‘antimicrobial’. Since most of the AMPs in positive dataset are with 10–80 amino acids, we randomly cut out a fragment with the same length range from each sequence and added them to the negative dataset. After eliminating those sequences with non-standard residues ‘B’, ‘J’, ‘O’, ‘U’, ‘X’, or ‘Z’, the final negative dataset thus obtained contained 10,014 non-AMP sequences.

#### Test set

CAMP [16] predicted dataset contained 1,153 sequences identified as antimicrobial based on the evidences of similarity or annotations in NCBI as ‘antimicrobial regions’ without exprerimental evidences. After eliminating those sequences containing non-standard residues ‘B’, ‘J’, ‘O’, ‘U’, ‘X’, or ‘Z’, 1,136 sequences were left that will serve as independent positive test dataset. As mentioned above, only 35 peptides are experimentally-verified no antimicrobial activity, and we had used these peptides as negative samples in the training dataset. Therefore, there were no more peptides left that could be used as independent negative samples for the test dataset in this study.

#### Cutoff threshold for sequence identity

Generally, homologous sequences in the datasets often influence the performance of the predictors. In order to remove the homologous peptides inside the training dataset and between the training and test datasets, a cutoff threshold of 70% was imposed to exclude those peptides from the training set that have equal to or greater than 70% sequence identity to any other in the training/test set by the CD-HIT program [29]. As a result, the training set thus obtained contained 9731 sequences, including 870 AMPs and 8661 non-AMPs.

It is known to us that the peptide's function is strongly related to its sequence order. Therefore we first apply the sequence alignment algorithm to predict AMPs. Secondly, we use amino acid composition and pseudo amino acid composition which can approximately reflect the sequence order [30], to deal with those peptides which can't be performed by the sequence alignment method.

### Sequence alignment method

Sequence alignment is a very important problem in Bioinformatics [31]. The sequences segments with high identify are inclined to share the structure and function. In the past decades, various sophisticated method such as FASTA, BLAST, HMMER and Smith-Waterman algorithm [32], [33], [34], [35] were developed for local and global alignments for DNA and protein sequences. Here, BLASTP [36] was used to predict AMPs, which can be described as follows. First, let us suppose a query peptide *P* and the training set 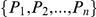, then the high-scoring segment pairs (HSPs) score between the query peptide and each peptide in the training set are calculated by BLASTP with default parameters. Then the peptide is predicted to share the same category as the peptide *P_k_* if the HSP score between *P* and *P_k_* is higher than other scores. Expressed in a formula, *P_k_* subjects to
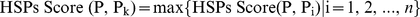
(1)If more than one *P_k_* fulfils the Eq. (1), one of them is chosen at random and its category was assigned to the query peptide *P*.

### Feature selection method

In this research, amino acid composition and pseudo-amino acid composition were used to code the AMP sequences.

#### Amino acid composition

Amino acid composition is a basic feature of protein sequence [25], which is closely correlated with its attributes, such as subcellular location [37], [38], [39], [40], [41], folding type [17], [42], secondary structure content [43], and domain [44]. Amino acid composition consists of 20 discrete numbers, each of which represents the occurrence frequency of the native amino acid in a protein sequence. Therefore, the protein can be coded into a 20-D (dimensional) numerical vector by the amino acid composition.

#### Pseudo-amino acid composition

The concept of pseudo-amino acid composition (PseAAC) was originally introduced by Chou for predicting the protein subcellular locations and membrane protein types [19]. Based on the conventional amino acid composition, Chou proposed a set of discrete numbers to take into account some sequence order effects. PseAAC has been proved to be an extremely effective feature in treating many protein and protein-related systems (see, e.g., [45], [46], [47], [48], [49], [50], [51], [52], [53], [54], [55], [56], [57], [58], [59], [60], [61], [62], [63], [64], [65], [66], [67], [68], [69], [70], [71], [72] as well as the Wikipedia web page at http://en.wikipedia.org/wiki/Pseudo_amino_acid_composition). For the detailed description about PseAAC, refer to [19], [73] and a recent comprehensive review [74]. Here, for reader's convenience, the concept of PseAAC is briefly described as follows.

Suppose a protein sequence of *L* amino acid residues:
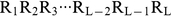
(2)The sequence order effect of the protein can be reflected by a set of discrete correlation factors, which are calculated as follows:
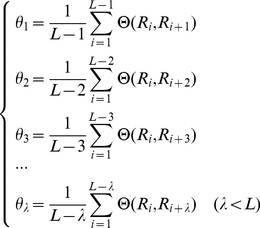
(3)where 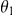, 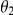, 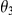, 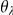 are the first-tier, second-tier, third-tier, 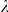-th tier correlation factors. And the correlation function is
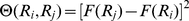
(4)where 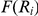 is the feature (e.g. hydrophilicity) value of the amino acid 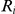. The value is converted from the original feature value of the amino acid according to the following equation:
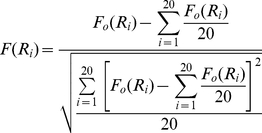
(5)where 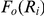 is the original feature value of the amino acid 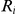. Thus, the PseAAC of a protein can be represented by a (20+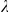)-D vector as follows:
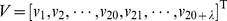
(6)where superscript **T** is the transpose operator and
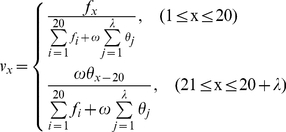
(7)where 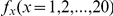 represent the occurrence frequencies of the 20 amino acids in the protein sequence, 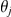 represents the *j-th* tier sequence correlation factor calculated according to Eq. (3), and 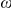 represents the weight for the sequence order effect. Based on the above description, we know that the first 20 components in Eq. (6) reflect the effect of the conventional amino acid composition, while the remaining 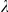 components are the correlation factors reflecting the effect of sequence order. A set of such 20+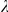 numbers is named PseAAC. In this study, we chose 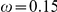 and 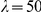 for getting the optimal results.

In this study, the codon diversity, electrostatic charge, molecular volume, polarity, and secondary structure are used to describe the physicochemical and biochemical properties of amino acids. And the values of the 5 features of the amino acids are retrieved from [20], [75], [76], as shown in **Table 1**. For each of the five features, a set of discrete correlation factors can be calculated according to Eq. (3) and Eq. (4) so as to contribute 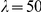 additional components for defining the protein sequence according to Eq. (6). Likewise, the similar approach can also be used to code the AMPs.

**Table 1:** The physicochemical and biochemical properties of the 20 amino acids.

Amino Acid	Polarity	Secondary structure	Molecular volume	Codon diversity	Electrostatic charge
A	−0.591	−1.302	−0.733	1.57	−0.146
C	−1.343	0.465	−0.862	−1.02	−0.255
D	1.05	0.302	−3.656	−0.259	−3.242
E	1.357	−1.453	1.477	0.113	−0.837
F	−1.006	−0.59	1.891	−0.397	0.412
G	−0.384	1.652	1.33	1.045	2.064
H	0.336	−0.417	−1.673	−1.474	−0.078
I	−1.239	−0.547	2.131	0.393	0.816
K	1.831	−0.561	0.533	−0.277	1.648
L	−1.019	−0.987	−1.505	1.266	−0.912
M	−0.663	−1.524	2.219	−1.005	1.212
N	0.945	0.828	1.299	−0.169	0.933
P	0.189	2.081	−1.628	0.421	−1.392
Q	0.931	−0.179	−3.005	−0.503	−1.853
R	1.538	−0.055	1.502	0.44	2.897
S	−0.228	1.399	−4.76	0.67	−2.647
T	−0.032	0.326	2.213	0.908	1.313
V	−1.337	−0.279	−0.544	1.242	−1.262
W	−0.595	0.009	0.672	−2.128	−0.184
Y	0.26	0.83	3.097	−0.838	1.512

Listed below are the scores of the physicochemical and biochemical properties of the 20 amino acids, each of which can be coded by a 5-dimensional vector.

Since each of the aforementioned five features (cf. **Table 1**) can generate 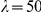 discrete numbers, the AMPs will be defined in a (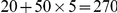)-D vector space.

In the feature space, we firstly prioritized the 270 features by the Maximum Relevance, Minimum Redundancy (mRMR) method. Based on the feature order, Incremental Feature Selection (IFS) method was employed to select the optimal feature subset. The prediction model was constructed according to Nearest Neighbor Algorithm (NNA) and evaluated by the jackknife test.

#### mRMR method

In pattern recognition, feature selection is an important procedure for constructing the classifier. Generally, a “good” feature for classification is considered to be not only highly correlated to the class, but also lowly redundant to the already selected features. Here, the Maximum Relevance, Minimum Redundancy [21] (mRMR) method was employed to sort the 270 features according to the descending order. The key ideas of the method are the Maximum Relevance criterion and Minimum Redundancy criterion as meant by its name. According to the Maximum Relevance criterion, the feature to be selected should have the maximal correlation with the class variable; while according to the Minimum Redundancy criterion, the feature to be selected should have minimal redundancy to the already selected features. Features are selected from the 270-D feature space one by one, being put into the MaxRel feature list by applying the Maximum Relevance criterion, and being put into the mRMR feature list by applying both the criteria. Both the relevance and redundancy are quantified by the mutual information (MI) defined as follows
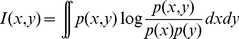
(8)where 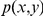 is the joint probabilistic density for feature x and feature y, 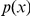 and 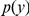 are the marginal probabilistic densities for feature x and feature y, respectively.

Suppose the whole feature set is denoted by Ω, the already selected feature set with *m* features by Ω_s_ and the feature set with *n* features by Ω_t_. The relevance *D* between the feature *f* in set Ω_t_ and the class *c* is calculated by
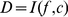
(9)The redundancy *R* of *f* with all the features in Ω_s_ is calculated by
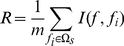
(10)To select the feature *f*_i_ in set Ω_t_ with the maximum relevance and minimum redundancy to already selected features in set Ω_s_, Eq. (9) and Eq. (10) are combined to generate the function:
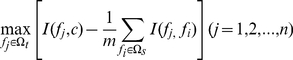
(11)Subsequently, the selected feature *f*_i_ will be taken away from the set Ω_t_ and added into the set Ω_s_. Such a process will be repeated until all the features are taken away from the set Ω_t_ and added into the set Ω_s_. The better the feature is, the earlier it will be selected.

#### Nearest Neighbor Algorithm

Nearest Neighbor Algorithm (NNA) [23] is a simple and effective instance-based learning method. It assigns the unknown sample to the class of the nearest neighbor. The distance function, the core of the algorithm, can be defined as follows [68]:
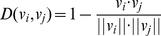
(12)where the symbol 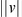 stands for the vector module of the sample, and 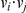 stands for the dot product of the two coding vectors.

Suppose a queried peptide with the 270-D coding vector 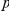 and the training set comprised of *n* classified peptides with the coding vector set 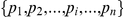 respectively. Then the queried peptides will be assigned to the class of vector 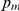, which satisfies
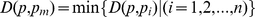
(13)If more than one 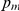 satisfies to Eq. (9), the class of one of these peptides will be randomly selected as the predicted result for the queried peptide.

#### Incremental Feature Selection

In essence, feature selection is a combinatorial optimization problem. Its goal is to seek the feature subset that maximizes the performance of the predictor. To find the optimal feature subset from the feature space with *N* features, all the combinations of *N* features should be tried from the point of view of the exhaustion principle, which is of computational intractability. Therefore Incremental Feature Selection [76], [77] (IFS) method was utilized to get the approximate solutions for this problem.

Based on features prioritized in the mRMR feature list, 270 feature subsets were obtained according to
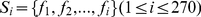
(14)where 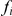 is the *i-th* feature in the mRMR feature list.

Then a NNA predictor was constructed for each feature subset and evaluated by the jackknife test. With the number of features of subset 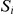 as its x-axis and accuracy as its y-axis, IFS curve was plotted to reveal the relation between the performance of the NNA predictor and the feature subset. The optimal feature subset is considered with the highest prediction accuracy, and the predictor thus obtained was used to classify the peptides.

### Overall prediction

For a query peptide, BLAST method was first applied to estimate whether it has antimicrobial activity. If it did not have any hits against the training sequences, then the Feature selection method was applied.

In statistical prediction, the following three cross-validation methods are often used to examine a predictor for its anticipated accuracy: independent dataset test, subsampling (K-fold cross-validation) test, and jackknife test [78]. In this study the jackknife test was adopted to examine the quality of the current predictor. During the jackknifing process, each of the peptide samples was in turn singled out from the benchmark dataset as a test sample, and identified by the prediction engine trained by the rest of the peptide samples in the dataset.

**Figure 1:**
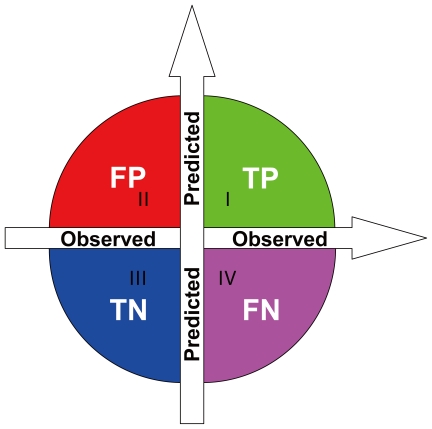
An illustration to show (I) TP (true positive) quadrant (green) for correct prediction of positive dataset, (II) FP (false positive) quadrant (red) for incorrect prediction of negative dataset; (III) TN (true negative) quadrant (blue) for correct prediction of negative dataset; and (IV) FN (false negative) quadrant (pink) for incorrect prediction of positive dataset.

The following equations were often used in literatures to reflect the prediction quality:
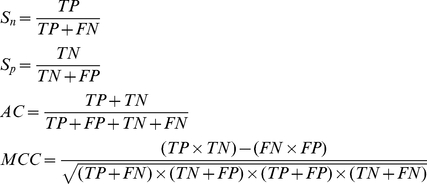
(15)where *S_n_* reflects the sensitivity, *S_p_* the specificity, *AC* the accuracy, and *MCC* the Mathews correlation coefficient; while *T*P represents the true positive, *TN*, the true negative; *FP*, the false positive, and *FN*, the false negative (**Figure 1**). *S_n_*, *S_p_* and *AC* stand for the success rates of prediction on positive, negative and overall datasets, respectively. *MCC* is used to evaluate the performance of the predictor when the positive and negative samples in the dataset are out-of-balance. Its value ranges from −1 to 1, and a larger *MCC* means a better prediction.

## Results and Discussion

### Results of sequence alignment method

In the jackknife cross-validation, each peptide was singled out from the benchmark data set as the query peptide, and the remaining peptides would serve as the training data set to train the predictor. Then the BLASTP method was applied to classify the peptide according to Eq. (1). However, some query peptides could not be processed by the method because no hits at all were found between them and the peptides in the training dataset. Among the 9731 peptides in the benchmark data set, 5855 peptides were predicted by the BLAST. The predicted results were shown in **Table 2**. The *S_n_*, *S_p_*, *AC*, and *MCC* were 91.22%, 95.55%, 95.12%, and 0.7723, respectively.

**Table 2:** The predicted results of the three methods.

Method	Number of Predicted Peptides	S_n_ (%)	S_p_ (%)	AC (%)	MCC
Sequence Alignment Method	5855	91.22	95.55	95.12	0.7723
Feature selection Method	3876	56.83	93.19	90.58	0.6426
Integrated Method	9731	80.23	94.59	93.31	0.7312

### Results of feature selection method

As the sequence alignment method could not deal with all the peptides, we designed the feature selection method to classify the remaining 3876 (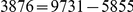) peptides.

**Figure 2:**
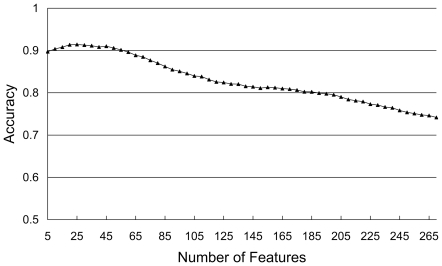
IFS curve. It reveals the relation between the performance of the NNA predictor and the feature subset. The IFS curve arrives at the apogee when the feature set is comprised of the first 25 features in the mRMR feature list.

Here, the prediction model was constructed as follows. All the peptides in the benchmark data set were firstly represented by the 270 features retrieved from the amino acid composition and pseudo-amino acid composition. The mRMR program (http://penglab.janelia.org/proj/mRMR/index.htm) was then applied to prioritize the features according to the Maximum Relevance criterion and Minimum Redundancy criterion. The MaxRel feature list and mRMR feature list thus obtained can be found in Table S1 and Table S2, respectively. Based on the sorted feature in mRMR feature list, the 270 feature subsets were constructed according to Eq. (14). Each of the feature subsets was used to recode the peptides in the dataset and construct the prediction model according to NNA. The prediction accuracies of the NNA predictor evaluated by jackknife test are shown in the IFS curve (**Figure 2**). It was observed that the peak of the accuracy was corresponding to the number of features at 25. Hence, the optimal feature subset was obtained with the first 25 features in the mRMR feature list. Therefore the predictor with these 25 features was used to cope with the 3876 peptides. The predicted results were also shown in **Table 2**. The *S_n_*, *S_p_*, *AC*, and *MCC* were 56.83%, 93.19%, 90.58%, and 0.6426, respectively.

### The overall predicted results

By combining the results of prediction from sequence alignment method and sequence based method, the overall success rates for the benchmark data set were obtained, as shown in **Table 2**. Evaluated by jackknife test, the *S_n_*, *S_p_*, *AC*, and *MCC* were 80.23%, 94.59%, 93.31%, and 0.7312, respectively, indicating a good prediction from the integration of the two methods. From the table, we can see that although BLASTP method obtained good predicted results, it could not deal with all the peptides. As a fall-back, the feature selection method was used to process the remaining peptides. By integrating the two methods, the hybrid one leads to satisfactory results.

### Independent test and comparison with the existing predictors

Generally speaking, the independent dataset is used for demonstrating how to use the predictor for practical applications [37]. This is because each of the peptides singled-out from the benchmark data set during the jackknifing process can actually be deemed as a sample of an independent data set. Now, just as a demonstration, let us use the benchmark dataset as a training dataset to identify the 1,136 AMP sequences collected in the independent dataset. The prediction sensitivity thus obtained with the integrated method was 72.27%, somewhat lower than the rate of jackknife test *S_n_*, this may because some AMPs in the test set were derived according to the annotations in NCBI based on the similarity principle and hence cannot avoid some sort of arbitrariness or false positive.

Up to now, several computational methods [10], [11], [12], [13], [14], [15], [16] have been proposed for the predicting AMPs. However, AMPer method [10] is not available at http://www.cnbi2.com/cgi-bin/amp.pl as described in [10]. BACTIBASE [11], [12] and PhytAMP [13] methods were specifically designed for bacteriocin and plant respectively. As for AntiBP [14] and AntiBP2 methods [15], they were designed for identifying the AMPs in a protein sequence, and hence could not be used to compare with our method. To make the comparison meaningful, our method was compared with CAMP method [16], which was developed based on the Random Forests (RF), SVM, and Discriminant Analysis (DA). In the comparison, the original 2,752 AMPs and 10,014 non-AMPs were treated as the training set. This is because to make the predictor better, nornally all the training samples need to be used. The comparison results are shown in the **Table 3**. The prediction *S_n_* by our method was 84.95%, higher than the predicted results of CAMP, indicating that our method outperformed CAMP.

**Table 3:** Comparison between CAMP and our method on the test set.

Method	Algorithm	Predicted AMPs	S_n_ (%)
CAMP	Support Vector Machine	866	76.23
CAMP	Random Forest	852	75.00
CAMP	Discriminant Analysis	881	77.55
Our Method	BLASTP+Nearest Neighbor Algorithm	965	84.95

### Comparison between sequence alignment method and feature selection method

In this study, sequence alignment method and feature selection method were developed to identify the AMPs from peptides. To compare the performance between them, each method was used alone to predict the peptides in the test set. To investigate the effect of sequence homology on the performance of the methods, original dataset (2,752 AMPs and 10,014 non-AMPs) and the dataset <0.7 sequence similarity were used. The predicted results are shown in **Table 4**.

**Table 4:** Comparison between sequence alignment method and feature selection method.

Dataset	Method	Number of Predicted Peptides	Number of Correctly Predicted Peptides	S_n_ (%)
Original Dataset with high sequence similarity	Sequence Alignment	986	896	90.87
	Feature Selection	1136	791	69.63
Dataset with <0.7 sequence similarity	Sequence Alignment	869	679	78.14
	Feature Selection	1136	692	60.92

From the table, we can see that the prediction *S_n_* by sequence alignment method is much higher than the *S_n_* by feature selection method. However, the sequence alignment could not deal with all the 1136 peptides in the test set. The sequence alignment method has the high predicted accuracies, while the feature selection method can predict all the peptides. To utilize the two advantages, the two methods were integrated to predict AMPs as above mentioned. The accuracies dropped by about 10% from the original dataset to dataset with <0.7 sequence similarity, which indicates sequence homology influenced the predictive quality.

### Analysis of optimal features

**Figure 3:**
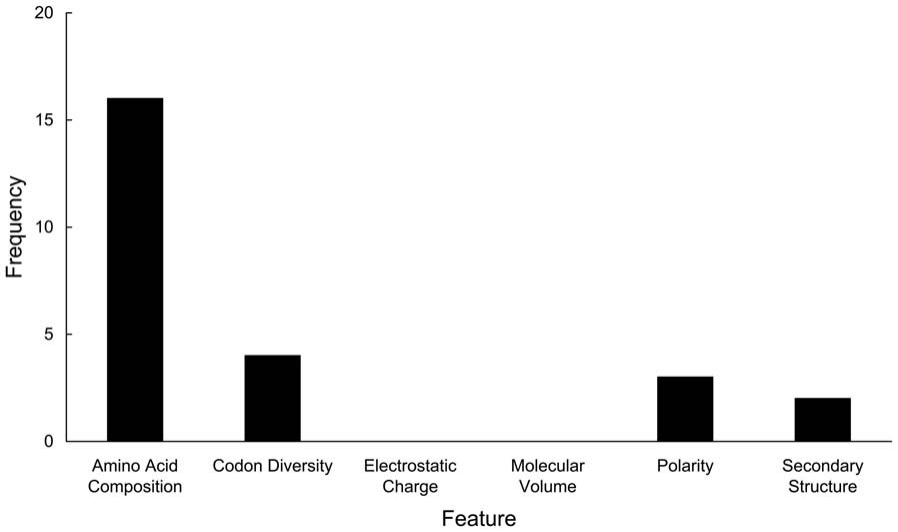
The numbers of each kind of features in optimal features. In the feature space, all the features can be classified into six kinds: amino acid composition, codon diversity, electrostatic charge, molecular volume, polarity and secondary structure.

**Figure 4:**
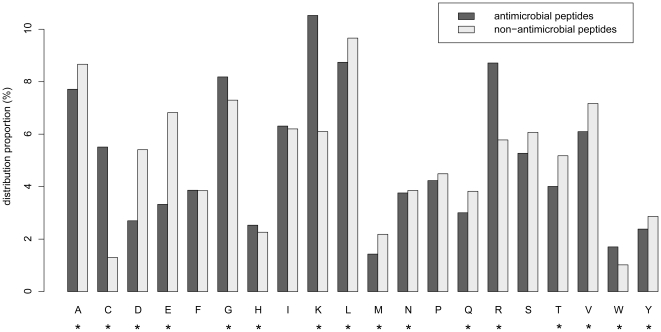
Amino acid distribution in AMPs and non-AMPs. * indicates amino acid in the optimal feature set.

Among the 25 optimal features obtained from the feature selection method, the one for the amino acid composition took up 64% (**Figure 3**). In the previous works, except for the simple and linear AMPs, larger AMPs are prone to contain certain amino acid types, such as cysteine, proline, arginine, tryptonphan, and histidine [79]. These five amino acids are all in our optimal features. Actually, according to our results, cysteine, arginine, tryptonphan and histidine are rich in antimicrobial peptides (**Figure 4**), fully consistent with the findings in [79], while proline is not obviously different between antimicrobial and non-antimicrobial peptides. Our results further confirm that amino acid composition is important for identify whether a peptide is an effector molecules of immunity. According to the ranks of these features, cysteine is the second one. Cysteine-rich peptides are particularly typical in plants [80], [81] and animals [82]. Pairs of cysteines forming intramolecular disulfide bridged are common in AMPs, thus allowing a complex three-dimensional structure, such as β-sheet [83] and β-turn [84]. Arginine, lysine and histidine are also important amino acid component features in our result. Arginine, lysine, and histidine in acidic environments are with positive net charged [85]. Meanwhile, the negative charged amino acids, glutamic acid and aspartic acid, are lack in AMPs (**Figure 4**). This may help AMPs to flip into biological membranes owing to the anionic phospholipid membranes [86]. Another AMP-rich amino acid is tryptophan. It is important for lipid binding [87], [88]and preferential in the protein-membrane interface [89]. The secondary structures, codon diversity as well as polarity of AMPs would ensure their abilities to defend microorganisms. All these effects may help AMPs disrupt the microbial membranes integrity.

### Conclusion

In this study, two methods are implemented: the sequence alignment method based on the BLASTP and the feature selection method with amino acid composition and pseudo amino acid composition features [90]. The prediction accuracy of the integrated method on the benchmark dataset is 80.23%. It is anticipated that the new method may be of use for helping to understand the role of peptide in antimicrobial activity, identify the natural AMPs, and design the synthetic AMPs against the resistance of microorganisms to antibiotics. For the convenience of readers, a user-friendly web-server is freely accessible at http://amp.biosino.org/.

## Supporting Information

Table S1 **The MaxRel feature list.**
(DOC)

Table S2 **The mRMR feature list.**
(DOC)
