# Supplementary material for: Prediction of Antimicrobial Peptides Based on Sequence Alignment and Feature Selection Methods
Source: PLoS One. 2011 Apr 13;6(4):e18476. doi: 10.1371/journal.pone.0018476 (PMC3076375; doi:10.1371/journal.pone.0018476)
Supplement: Table S2 — The mRMR feature list. (DOC) [file pone.0018476.s002.doc]

**Table S2: the mRMR feature list**

Listed below are the 270 features prioritized by mRMR method according to Maximum Relevance criterion and Minimum Redundancy criterion.

| Order | Feature |
| --- | --- |
| 1 | Amino Acid Composition (E) |
| 2 | Amino Acid Composition (C) |
| 3 | Amino Acid Composition (K) |
| 4 | Amino Acid Composition (M) |
| 5 | Amino Acid Composition (D) |
| 6 | Amino Acid Composition (V) |
| 7 | Polarity |
| 8 | Amino Acid Composition (R) |
| 9 | Amino Acid Composition (Q) |
| 10 | Amino Acid Composition (Y) |
| 11 | Amino Acid Composition (N) |
| 12 | Amino Acid Composition (H) |
| 13 | Codon Diversity |
| 14 | Amino Acid Composition (L) |
| 15 | Secondary Structure |
| 16 | Amino Acid Composition (T) |
| 17 | Secondary Structure |
| 18 | Polarity |
| 19 | Amino Acid Composition (A) |
| 20 | Codon Diversity |
| 21 | Amino Acid Composition (W) |
| 22 | Amino Acid Composition (G) |
| 23 | Polarity |
| 24 | Codon Diversity |
| 25 | Codon Diversity |
| 26 | Secondary Structure |
| 27 | Molecular Volume |
| 28 | Molecular Volume |
| 29 | Polarity |
| 30 | Molecular Volume |
| 31 | Amino Acid Composition (I) |
| 32 | Codon Diversity |
| 33 | Amino Acid Composition (P) |
| 34 | Codon Diversity |
| 35 | Secondary Structure |
| 36 | Electrostatic Charge |
| 37 | Polarity |
| 38 | Codon Diversity |
| 39 | Secondary Structure |
| 40 | Polarity |
| 41 | Codon Diversity |
| 42 | Molecular Volume |
| 43 | Secondary Structure |
| 44 | Amino Acid Composition (S) |
| 45 | Amino Acid Composition (F) |
| 46 | Polarity |
| 47 | Secondary Structure |
| 48 | Codon Diversity |
| 49 | Molecular Volume |
| 50 | Secondary Structure |
| 51 | Polarity |
| 52 | Molecular Volume |
| 53 | Codon Diversity |
| 54 | Polarity |
| 55 | Electrostatic Charge |
| 56 | Secondary Structure |
| 57 | Secondary Structure |
| 58 | Codon Diversity |
| 59 | Molecular Volume |
| 60 | Molecular Volume |
| 61 | Polarity |
| 62 | Polarity |
| 63 | Electrostatic Charge |
| 64 | Secondary Structure |
| 65 | Electrostatic Charge |
| 66 | Electrostatic Charge |
| 67 | Secondary Structure |
| 68 | Polarity |
| 69 | Molecular Volume |
| 70 | Electrostatic Charge |
| 71 | Molecular Volume |
| 72 | Electrostatic Charge |
| 73 | Codon Diversity |
| 74 | Electrostatic Charge |
| 75 | Secondary Structure |
| 76 | Electrostatic Charge |
| 77 | Electrostatic Charge |
| 78 | Polarity |
| 79 | Molecular Volume |
| 80 | Secondary Structure |
| 81 | Codon Diversity |
| 82 | Molecular Volume |
| 83 | Codon Diversity |
| 84 | Polarity |
| 85 | Codon Diversity |
| 86 | Molecular Volume |
| 87 | Secondary Structure |
| 88 | Polarity |
| 89 | Electrostatic Charge |
| 90 | Codon Diversity |
| 91 | Electrostatic Charge |
| 92 | Molecular Volume |
| 93 | Electrostatic Charge |
| 94 | Secondary Structure |
| 95 | Secondary Structure |
| 96 | Codon Diversity |
| 97 | Polarity |
| 98 | Secondary Structure |
| 99 | Polarity |
| 100 | Codon Diversity |
| 101 | Secondary Structure |
| 102 | Electrostatic Charge |
| 103 | Secondary Structure |
| 104 | Secondary Structure |
| 105 | Molecular Volume |
| 106 | Polarity |
| 107 | Polarity |
| 108 | Electrostatic Charge |
| 109 | Electrostatic Charge |
| 110 | Molecular Volume |
| 111 | Molecular Volume |
| 112 | Polarity |
| 113 | Codon Diversity |
| 114 | Molecular Volume |
| 115 | Electrostatic Charge |
| 116 | Codon Diversity |
| 117 | Molecular Volume |
| 118 | Secondary Structure |
| 119 | Codon Diversity |
| 120 | Molecular Volume |
| 121 | Electrostatic Charge |
| 122 | Codon Diversity |
| 123 | Secondary Structure |
| 124 | Molecular Volume |
| 125 | Polarity |
| 126 | Polarity |
| 127 | Codon Diversity |
| 128 | Molecular Volume |
| 129 | Codon Diversity |
| 130 | Electrostatic Charge |
| 131 | Electrostatic Charge |
| 132 | Polarity |
| 133 | Electrostatic Charge |
| 134 | Polarity |
| 135 | Codon Diversity |
| 136 | Electrostatic Charge |
| 137 | Polarity |
| 138 | Secondary Structure |
| 139 | Secondary Structure |
| 140 | Molecular Volume |
| 141 | Molecular Volume |
| 142 | Polarity |
| 143 | Codon Diversity |
| 144 | Secondary Structure |
| 145 | Secondary Structure |
| 146 | Electrostatic Charge |
| 147 | Electrostatic Charge |
| 148 | Polarity |
| 149 | Secondary Structure |
| 150 | Molecular Volume |
| 151 | Electrostatic Charge |
| 152 | Electrostatic Charge |
| 153 | Secondary Structure |
| 154 | Molecular Volume |
| 155 | Secondary Structure |
| 156 | Polarity |
| 157 | Molecular Volume |
| 158 | Polarity |
| 159 | Codon Diversity |
| 160 | Codon Diversity |
| 161 | Electrostatic Charge |
| 162 | Electrostatic Charge |
| 163 | Polarity |
| 164 | Secondary Structure |
| 165 | Codon Diversity |
| 166 | Molecular Volume |
| 167 | Molecular Volume |
| 168 | Codon Diversity |
| 169 | Molecular Volume |
| 170 | Electrostatic Charge |
| 171 | Secondary Structure |
| 172 | Electrostatic Charge |
| 173 | Molecular Volume |
| 174 | Polarity |
| 175 | Codon Diversity |
| 176 | Codon Diversity |
| 177 | Electrostatic Charge |
| 178 | Secondary Structure |
| 179 | Molecular Volume |
| 180 | Polarity |
| 181 | Secondary Structure |
| 182 | Electrostatic Charge |
| 183 | Codon Diversity |
| 184 | Molecular Volume |
| 185 | Polarity |
| 186 | Secondary Structure |
| 187 | Electrostatic Charge |
| 188 | Polarity |
| 189 | Molecular Volume |
| 190 | Codon Diversity |
| 191 | Secondary Structure |
| 192 | Codon Diversity |
| 193 | Electrostatic Charge |
| 194 | Polarity |
| 195 | Molecular Volume |
| 196 | Secondary Structure |
| 197 | Electrostatic Charge |
| 198 | Codon Diversity |
| 199 | Electrostatic Charge |
| 200 | Molecular Volume |
| 201 | Polarity |
| 202 | Secondary Structure |
| 203 | Codon Diversity |
| 204 | Electrostatic Charge |
| 205 | Electrostatic Charge |
| 206 | Codon Diversity |
| 207 | Electrostatic Charge |
| 208 | Polarity |
| 209 | Codon Diversity |
| 210 | Polarity |
| 211 | Molecular Volume |
| 212 | Molecular Volume |
| 213 | Electrostatic Charge |
| 214 | Secondary Structure |
| 215 | Secondary Structure |
| 216 | Molecular Volume |
| 217 | Molecular Volume |
| 218 | Codon Diversity |
| 219 | Polarity |
| 220 | Polarity |
| 221 | Polarity |
| 222 | Secondary Structure |
| 223 | Codon Diversity |
| 224 | Secondary Structure |
| 225 | Secondary Structure |
| 226 | Codon Diversity |
| 227 | Secondary Structure |
| 228 | Electrostatic Charge |
| 229 | Molecular Volume |
| 230 | Electrostatic Charge |
| 231 | Electrostatic Charge |
| 232 | Codon Diversity |
| 233 | Electrostatic Charge |
| 234 | Polarity |
| 235 | Molecular Volume |
| 236 | Secondary Structure |
| 237 | Electrostatic Charge |
| 238 | Molecular Volume |
| 239 | Electrostatic Charge |
| 240 | Polarity |
| 241 | Molecular Volume |
| 242 | Molecular Volume |
| 243 | Secondary Structure |
| 244 | Secondary Structure |
| 245 | Polarity |
| 246 | Secondary Structure |
| 247 | Polarity |
| 248 | Secondary Structure |
| 249 | Polarity |
| 250 | Codon Diversity |
| 251 | Molecular Volume |
| 252 | Molecular Volume |
| 253 | Codon Diversity |
| 254 | Codon Diversity |
| 255 | Codon Diversity |
| 256 | Molecular Volume |
| 257 | Electrostatic Charge |
| 258 | Codon Diversity |
| 259 | Electrostatic Charge |
| 260 | Codon Diversity |
| 261 | Molecular Volume |
| 262 | Polarity |
| 263 | Polarity |
| 264 | Molecular Volume |
| 265 | Polarity |
| 266 | Polarity |
| 267 | Secondary Structure |
| 268 | Codon Diversity |
| 269 | Electrostatic Charge |
| 270 | Electrostatic Charge |
